# Activation of sirtuin 1/3 improves vascular hyporeactivity in severe hemorrhagic shock by alleviation of mitochondrial damage

**DOI:** 10.18632/oncotarget.6076

**Published:** 2015-10-12

**Authors:** Pengyun Li, Xianzhong Meng, Huining Bian, Nana Burns, Ke-seng Zhao, Rui Song

**Affiliations:** ^1^ Guangdong Key Laboratory of Shock and Microcirculation Research, Department of Pathophysiology, Southern Medical University, Guangzhou, China; ^2^ Key Laboratory of Medical Electrophysiology, and Institute of Cardiovascular Research, Sichuan Medical University, Luzhou, China; ^3^ Department of Surgery, University of Colorado Denver, Aurora, CO, USA; ^4^ Department of Burns, Guangdong General Hospital, Guangdong Academy of Medical Sciences, Guangzhou, China; ^5^ Department of Pediatrics, University of Colorado Denver, Aurora, CO, USA

**Keywords:** vasoreactivity, sirtuin, deacetylation, mitochondria, hemorrhagic shock, Pathology Section

## Abstract

Vascular hyporeactivity is one of the major causes responsible for refractory hypotension and associated mortality in severe hemorrhagic shock. Mitochondrial permeability transition (mPT) pore opening in arteriolar smooth muscle cells (ASMCs) is involved in the pathogenesis of vascular hyporeactivity. However, the molecular mechanism underlying mitochondrial injury in ASMCs during hemorrhagic shock is not well understood. Here we produced an *in vivo* model of severe hemorrhagic shock in adult Wistar rats. We found that sirtuin (SIRT)1/3 protein levels and deacetylase activities were decreased in ASMCs following severe shock. Immunofluorescence staining confirmed reduced levels of SIRT1 in the nucleus and SIRT3 in the mitochondria, respectively. Acetylation of cyclophilin D (CyPD), a component of mPT pore, was increased. SIRT1 activators suppressed mPT pore opening and ameliorated mitochondrial injury in ASMCs after severe shock. Furthermore, administration of SIRT1 activators improved vasoreactivity in rats under severe shock. Our data suggest that epigenetic mechanisms, namely histone post-translational modifications, are involved in regulation of mPT by SIRT1/SIRT3- mediated deacetylation of CyPD. SIRT1/3 is a promising therapeutic target for the treatment of severe hemorrhagic shock.

## INTRODUCTION

Severe or irreversible hemorrhagic shock is a life-threatening situation associated with significant morbidity and mortality directly due to massive blood loss or occurs indirectly to secondary multiple organ failure. Persistent low perfusion and refractory hypotension are the two major events associated with the pathogenesis of irreversible shock [1]. Previous studies have shown that ATP depletion in severe shock not only results from insufficient delivery of nutrients and oxygen, but also from cytopathic hypoxia involving damage to and dysfunction of mitochondria in arteriolar smooth muscle cells (ASMCs) [2, 3]. Growing evidence suggests that mitochondrial dysfunction plays a key role in the pathogenesis of tissue ischemia and reperfusion injury [4-6]. However, the cellular and molecular mechanisms of mitochondria-derived cytopathic hypoxia underlying the progression of refractory hypotension during severe shock remain elusive.

Recently, histone deacetylase sirtuin 1 (SIRT1) emerged as a crucial regulator of mitochondrial biogenesis and function [7, 8]. Mammalian SIRT1 is homologous to the yeast silent information regulator 2 and belongs to class III histone deacetylates including seven sirtuins (SIRT1-7). Sirtuins have unique functions due to their structure and specific subcellular location. SIRT1 is mainly localized to the nucleus, but is also found in other cellular compartments, such as the cytosol and the mitochondria [9]. The pharmaceutical agent SRT1720 has been reported to activate SIRT1, resulting in PGC-1α activation, increased mitochondrial fatty acid oxidation in the muscle, and improved running performance, muscle strength and coordination [10]. However, overexpression of SIRT1 decreases PGC-1α acetylation, PGC-1α activity, and mitochondrial proteins levels in C2C12 myotubes [11]. Resveratrol (Res), a SIRT1 activator, has been shown to restore SIRT1 activity and mitochondrial complex function in the liver, kidney and heart during injury or stress [12-14].

SIRT3 is a major mitochondrial deacetylase that regulates mitochondrial metabolism. Mitochondrial proteins are hyperacetylated in SIRT3 knockout mice, but not in SIRT4 or SIRT5 knockout mice [15]. It has been demonstrated that cyclophilin D (CyPD), which is localized to the mitochondrial matrix, modulates the mitochondrial permeability transition (mPT) pore opening [16, 17]. SIRT3-induced inactivation of CyPD by deacetylation is necessary for mitochondrial function in cancer cells [18]. Based on these data, we hypothesized that SIRT 1/3 may play an important role in the protection against hemorrhagic shock injury by modulating mitochondrial function. To test our hypothesis, we determined whether the expression and activity of SIRT1 and SIRT3 were altered during severe shock, and investigated the role of SIRT1 and SIRT3 in the regulation of mitochondrial permeability transition and mitochondrial ATP production. Finally, we evaluated the protective effects of SIRT1 and SIRT3 against vascular hyporeactivity and refractory hypotension during severe hemorrhagic shock.

## RESULTS

### Mitochondrial injury and mPT pore opening in ASMCs are associated with down-regulation of both SIRT1 and SIRT3 during severe hemorrhagic shock

**Figure 1:**
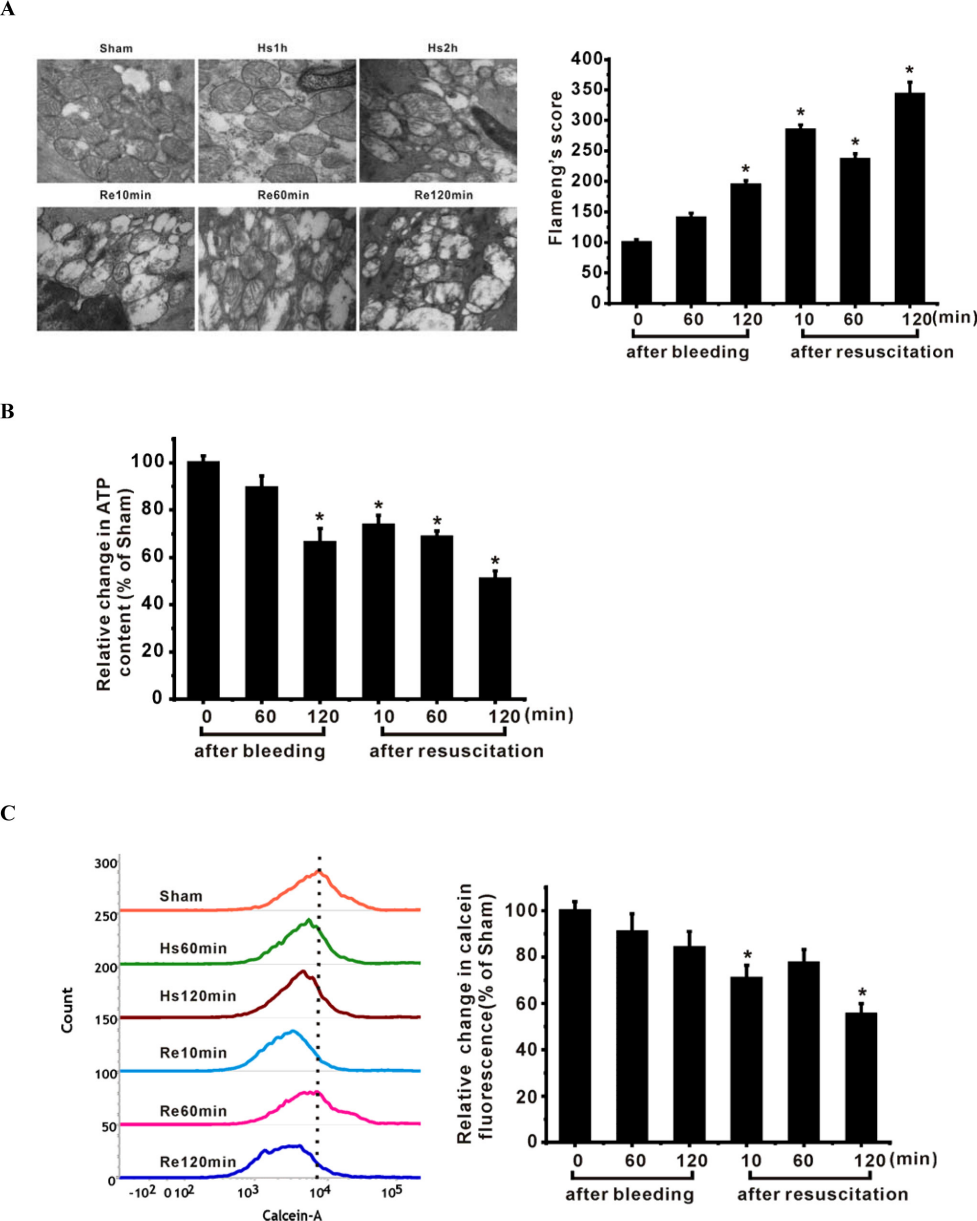

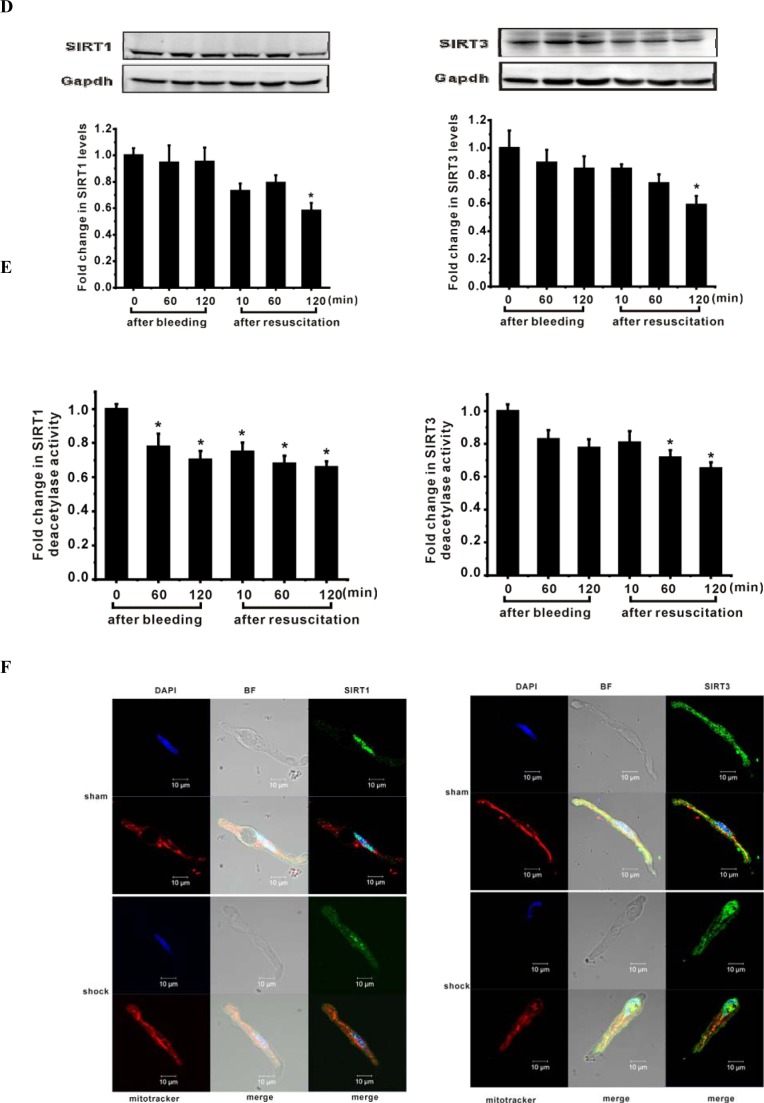

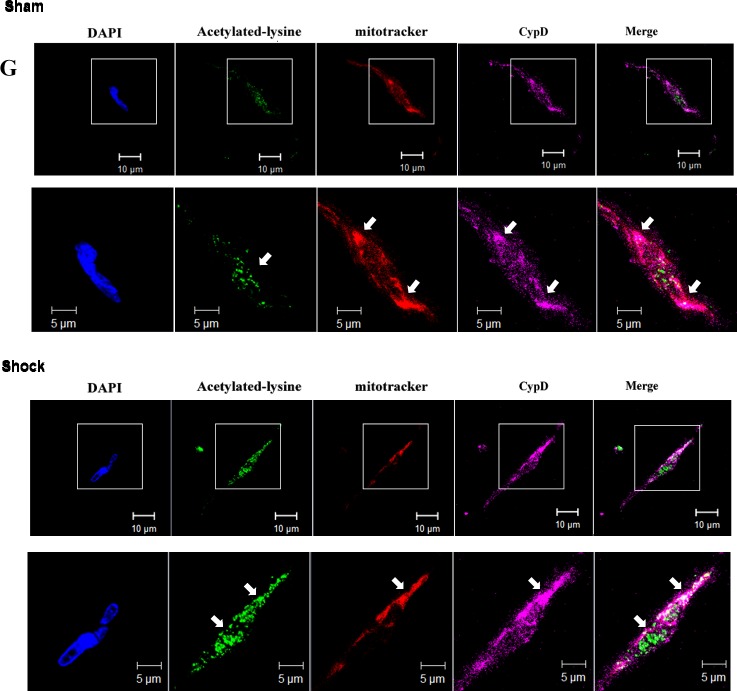
Mitochondrial injury in ASMCs of shock animals is associated with down-regulation of SIRT1/3 **A**. Representative transmission electron microscopic images show progressive mitochondria swelling with cristae disruption (50,000× magnification). Flameng's score analysis confirmed mitochondrial swelling during severe shock. **B**. ATP levels are decreased in ASMCs at different time points during hemorrhagic shock. **C**. MPT pore alterations during severe shock are analyzed by staining the cells with calcein-AM/CoCl2. A shift of the curves to the left indicates progressive decrease in the mean fluorescence intensity (MFI) of calcein. **D**. MFI of calcein is decreased especially at the time points of 10 min and 120 min after resuscitation in severe shock, suggesting the opening of mPT pore at the onset of resuscitation. **E**. SIRT1/3 protein expression and activities are reduced in mesenteric artery following severe shock. Representative immunoblots of 4 separated experiments and quantitative analysis showed that the expression of SIRT1 and SIRT3 was decreased in ASMCs. **F**. Deacetylase activities of SIRT1 and SIRT3 are decreased in ASMCs during severe shock. Representative immunofluorescence staining images show that SIRT1 is located in the nucleus of ASMCs but nuclear localization of SIRT1 is decreased following severe shock. The localization of SIRT3 in the mitochondria is decreased following severe shock. **G**. Representative immunofluorescence staining images show that acetylation of CyPD (white) is increased in ASMCs following severe shock. Scale bar = 10 μm. n = 6 or 7; *P < 0.05 vs. sham group. Hs=hemorrhagic shock; Re=resuscitation.

To determine the changes in mitochondrial phenotype and function during severe hemorrhagic shock, we examined mitochondrial ultrastructural morphology and ATP production in ASMCs isolated from rats with severe hemorrhagic shock. We found partly fragmentation, disorganization/disorientation, clumping, and/or loss of mitochondrial cristae at 2 h following hemorrhage. After resuscitation, we could not observe normal cristae in the longitudinal/linear pattern and found swollen mitochondria, instead (Figure 1A). Flameng score analysis showed significant mitochondrial injury in the ASMCs at 10 min and 2 h after resuscitation (Figure 1A). After resuscitation, ATP levels were decreased in ASMCs in a time-dependent manner (Figure 1B). Furthermore, calcein-AM quenching assay showed calcein fluorescence inflow following mPT pore opening in a time-dependent fashion, especially at 10 min and 2 h after resuscitation (Figure 1C).

We determined protein levels and deacetylase activity of SIRT1 and SIRT3 in ASMCs during severe shock. Western blot analysis showed that SIRT1 and SIRT3 protein levels were reduced during severe hemorrhagic shock (Figure 1D). In parallel with decreased SIRT1 and SIRT3 levels, the deacetylase activities of SIRT1 and SIRT3 were decreased (Figure 1E). Furthermore, immunofluorescence analysis showed that endogenous SIRT1 was located mostly in the nucleus, but nuclear localization of SIRT1 was decreased after 2 h of resuscitation (Figure 1F). Similarly, SIRT3 levels in the mitochondria were reduced in ASMCs isolated from shock animals (Figure 1F). Further, we found that acetylation of CyPD was increased in ASMCs of shock animals (Figure 1G).

### SIRT1/SIRT3 suppresses mPT pore opening and mitochondrial injury in ASMCs during shock

**Figure 2:**
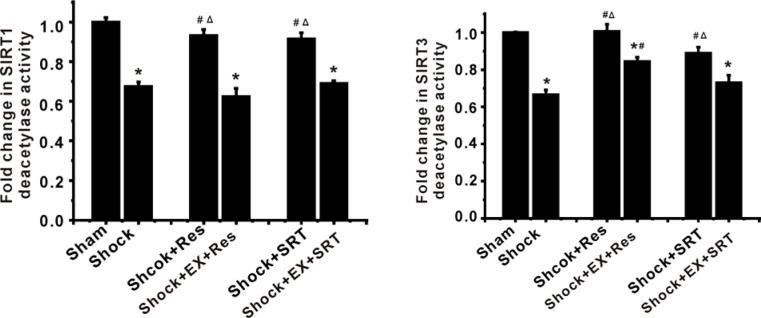
Modulation of SIRT1 activity leads to changes in SIRT3 deacetylase activity in ASMCs during severe shock **A.** The shock-associated reduction of SIRT1 deacetylase activity, as assessed by a commercially available fluorometric assay, is inhibited by resveratrol (Res) and SRT1720, a potent SIRT1 activator. EX 527, a selective SIRT1 inhibitor, inhibits the effects of Res and SRT1720. **B.** Res and SRT1720 enhance SIRT3 deacetylase activity in ASMCs during severe shock. Inhibition of SIRT1 activity suppresses the effects of Res and SRT1720 on SIRT3 deacetylase activity. *n* = 4; ^*^P < 0.05 *vs*. sham group; ^#^*P* < 0.05 *vs*. shock group; ^Δ^*P* < 0.05 in shock +Res group or shock +SRT1720 group *vs*. shock +Ex527+Res group or shock +Ex527 + SRT1720 group.

**Figure 3:**
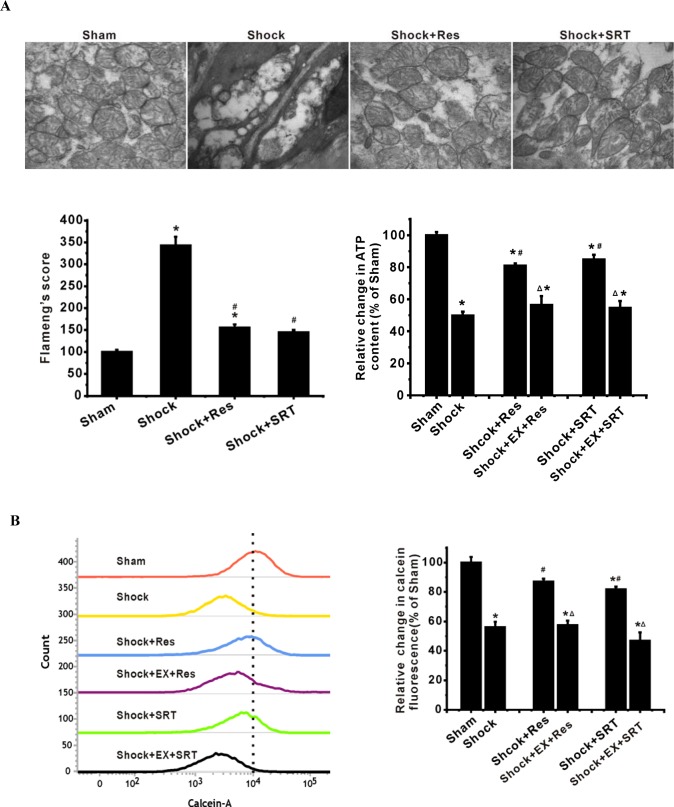

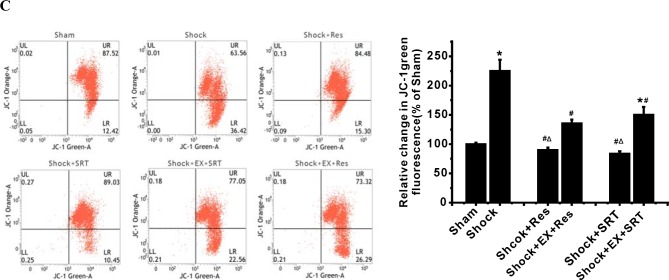
SIRT1/3 regulates mitochondrial energy production machinery by modulation of mPT in ASMCs during severe shock **A.** Activation of SIRT1/3 by resveratrol (Res) and SRT1720 suppresses the shock-associated mitochondrial injury and decrease in ATP production. Inhibition of SIRT1/3 activity enhances mitochondrial phenotype and functional damage. **B.** Mitochondrial membrane potential (MMP) and PTP opening are analyzed by flow cytometry with JC-1 and calcein-AM/COCl_2_, respectively. Representative FCM images and quantification data show that Res and SRT1720 suppress the shock-associated ΔΨm depolarization. **C.** Bar graph shows that Res and SRT1720 enhance the calcein fluorescence decreased by shock. *n* = 4 or 5; ^*^P < 0.05 *vs*. sham group; ^#^*P* < 0.05 *vs*. shock group; ^Δ^*P* < 0.05 shock + Res *vs*. shock +EX527+Res group; shock +SRT1720 group *vs*. shock +EX527+SRT1720 group.

To further determine the role of SIRT1/3 in mitochondrial phenotype and function, we exposed ASMCs to resveratrol (Res) and SRT1720, potent SIRT1 activators. We found that Res and SRT1720 relieved shock-caused reduction of SIRT1 deacetylase activity as assessed by fluorometric assay (Figure 2). However, the inhibition of SIRT1 activity by EX 527 suppressed the effects of Res and SRT1720 on deacetylase activities of SIRT1 and SIRT3 (Figure 2). Importantly, activation of SIRT1/3 by Res and SRT1720 attenuated shock-induced mitochondrial injury (Figure 3A) and decrease in ATP production (Figure 3A). In contrast, inhibition of SIRT1/3 activity enhanced mitochondrial damage (Figure 3A).

Opening of mPT pore results in subsequent swelling of mitochondria due to the influx of water and ions, leading to the dissipation of mitochondrial membrane potential (ΔΨm), uncoupling of oxidative phosphorylation and loss of ATP [20]. To better understand the protective effects of SIRT1 and SIRT3 on the mitochondria after shock, we determined the effects of Res and SRT1720 on mPT pore opening and ΔΨm in ASMCs. We found that Res and SRT1720 suppressed the shock-associated mPT pore opening and ΔΨm depolarization, while the inhibition of SIRT1/3 activity promoted mPT pore opening and ΔΨm depolarization after shock (Figure 3B and 3C). These results suggest that SIRT1/SIRT3 regulates mitochondrial phenotype and function primarily through the inhibition of mPT pore opening.

### SIRT1 activation improves microcirculation and mean arterial blood pressure (MAP) during shock

**Figure 4:**
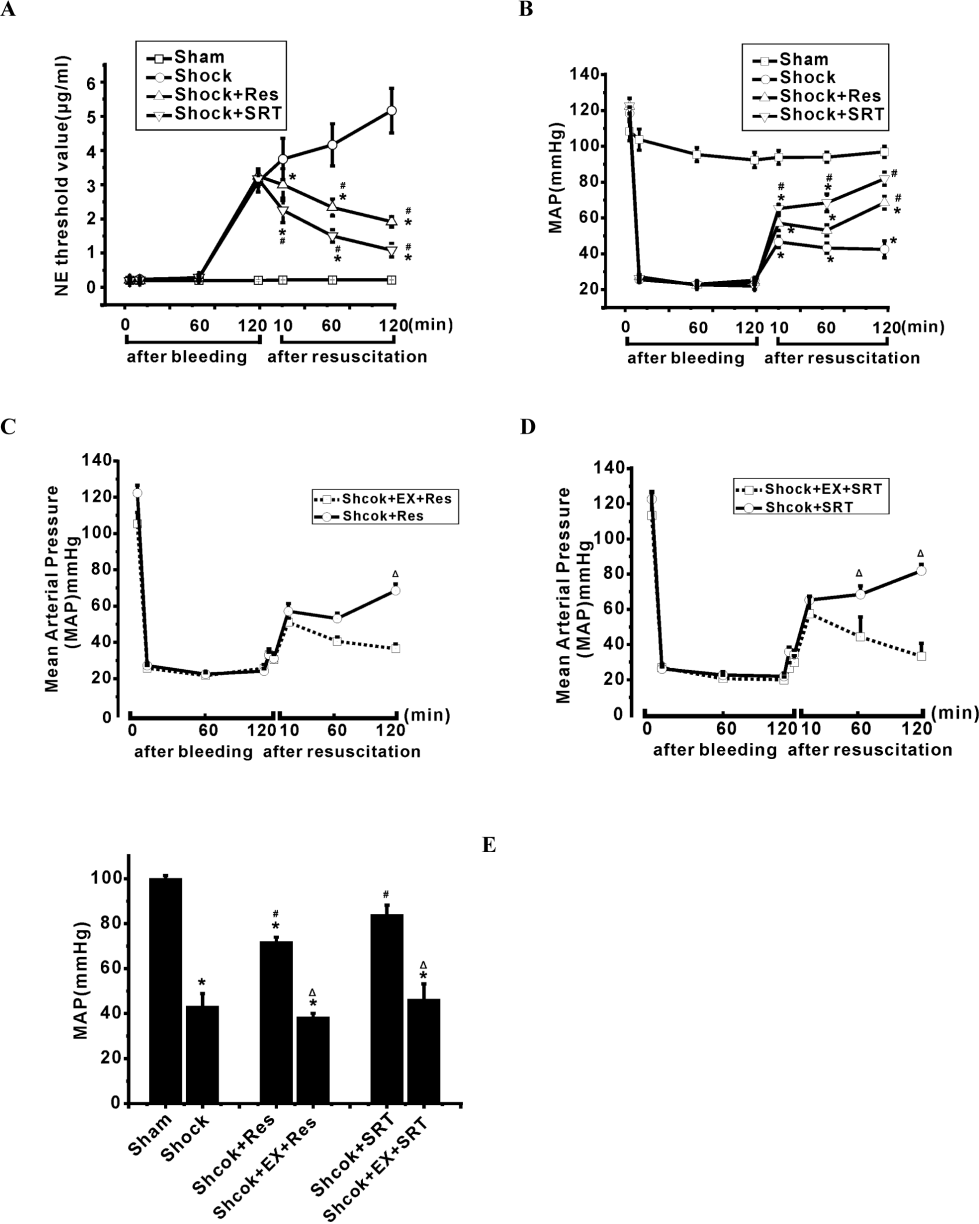
Modulation of SIRT1/3 regulates vasoreactivity and mean arterial pressure in the rats during severe shock Vasoreactivity and mean arterial pressure are measured before bleeding and at the end of a period including 2 h post-hemorrhage with or without different treatments. **A.** and **B.** Activation of SIRT1/3 by resveratrol (Res) and SRT1720 restores low vasoreactivity and improves the hypotension following severe shock. **C. D.** and **E.** Inhibition of SIRT1/3 activity relieves hypotension during severe shock. *n* = 5 or 6; ^*^P < 0.05 *vs*. sham group; ^#^*P* <0.05 *vs*. shock group; ^Δ^*P* < 0.05, shock+Res group *vs*. shock+EX527+Res group; shock+SRT1720 group *vs*. shock+EX527+SRT1720 group.

Our previous study demonstrated that mitochondrial injury in vascular cells plays an important role in the impaired vasoreactivity and microcirculation during shock [2]. To determine whether SIRT1/SIRT3 have protective effects in improving microcirculation, MAP and survival after severe hemorrhagic shock, we examined the effects of Res and SRT1720 on NE threshold concentration and MAP in the rats during severe shock. We found that Res decreased NE concentration from 5.69±1.67 μg/ml to 2.08±0.24 μg/ml but increased MAP from 42.51±4.69 mmHg to 68.57±3.64 mmHg at 2 h after resuscitation (Figure 4A). Similarly, SRT1720 decreased NE concentration to 1.20±0.46 μg/ml but increased MAP to 81.91±3.62 mmHg (Figure 4A). Furthermore, EX 527 suppressed the effects of Res and SRT1720 in improving low vasoreactivity and hypotension (Figure 4B). These data suggest that the activation of SIRT1 protects against low vasoreactivity and hypotension *in vivo* during severe shock.

## DISCUSSION

Severe or irreversible hemorrhagic shock causes high morbidity [21]. Our recent studies have demonstrated that mitochondrial injury and dysfunction in ASMCs play an important role in the pathogenesis of severe hemorrhagic shock. Regulation of mitochondrial biogenesis has recently emerged as a potential therapeutic strategy for the amelioration of ischemic cardiovascular diseases [22, 23]. In this study, we found that decreased levels and deacetylase activity of SIRT1/3 in mesenteric arteriolar SMCs of shock animals promote persistent opening of mPT pore and mitochondrial injury. Activation of SIRT1/3 by Res or SRT1720 restores SIRT1/3 activity, and attenuated mitochondrial injury. Importantly, administration of SIRT1 activator at 2 h post bleeding improves vasoresponse to noradrenaline. Taken together, our findings suggest that SIRT1/3 play a protective role during severe hemorrhagic shock.

Hemorrhagic shock causes global ischemic stress [24]. Much of the tissue damage following hemorrhagic shock is a result of hypoperfusion (during bleeding) and the restoration of perfusion (following resuscitation) [25]. During the compensatory stage (shock 10-60 min), various protection mechanisms in the body respond to the decline of blood volume, and there is no significant damage in the mitochondrial ultrastructure and function. However, autoregulation may be temporary during the process of severe shock when blood pressure is lower than a threshold for a long time. The period of 50-70 min most likely corresponds to the turning point from compensatory to decompensated stage [26], since mPT pore opening, mitochondrial swelling and cristae disruption are observed at 2 h after bleeding and at 2 h after resuscitation. Similarly, intracellular ATP level is progressively decreased, especially at 2 h after resuscitation. In accordance with our previous studies, the present study provides further evidence in support of the notion that mitochondria injury plays an important role in the pathogenesis of hemorrhagic shock.

The dissection of the pathways that regulate mitochondrial biogenesis and energy metabolism provides new insights into the mechanisms of mitochondrial damage in cardiovascular diseases [27, 28]. Histone deacetylases have been demonstrated to dramatically improve survival following lethal hemorrhagic shock in the rat [14, 29]. Recently, it has been reported that the activity of SIRT1/3 deacetylase is correlated with mitochondrial function [8]. In the present study, we investigated whether changes in SIRT1/3 levels and activity contribute to the severity of hemorrhagic shock. Our results revealed significant decreases in SIRT1 and SIRT3 levels and activity following hemorrhagic shock and indicate that such changes in SIRT1/3 worsens hemorrhagic shock by exacerbating mitochondria injury in vascular smooth muscle cells. These findings are consistent with previous reports on myocardial injury following ischemia-reperfusion [30, 31].

In agreement with most studies, we observed that SIRT1 is present both in the nucleus and in the cytoplasm with dominant expression in the nucleus [32]. SIRT3 is localized both in the nucleus and in the mitochondria [33, 34]. However, both nuclear localization of SIRT1 and mitochondrial localization of SIRT3 are reduced in ASMCs during hemorrhagic shock. Altered mPT is a key mechanism of ischemia-reperfusion injury and severe shock [20, 35, 36], and CypD is an important regulator of mPT pore [17, 37]. Recently, it was found that posttranslational modification of mitochondrial proteins *via* the acetylation/deacetylation of protein lysine residues plays an important role in the regulation of mitochondrial function [19, 38]. SIRT3 is the most robust mitochondrial deacetylase and its activity is necessary to prevent mitochondrial dysfunction by modulating the acetylation and activity of CyPD. Thus, the deacetylation of CyPD may be a control point for mPT pore regulation [15]. We found that acetylation of CyPD is enhanced in ASMCs following severe shock. To our knowledge, this is the first study to demonstrate acetylation-related epigenetic regulation of SIRT1/3 in the mitochondria during severe shock.

**Figure 5:**
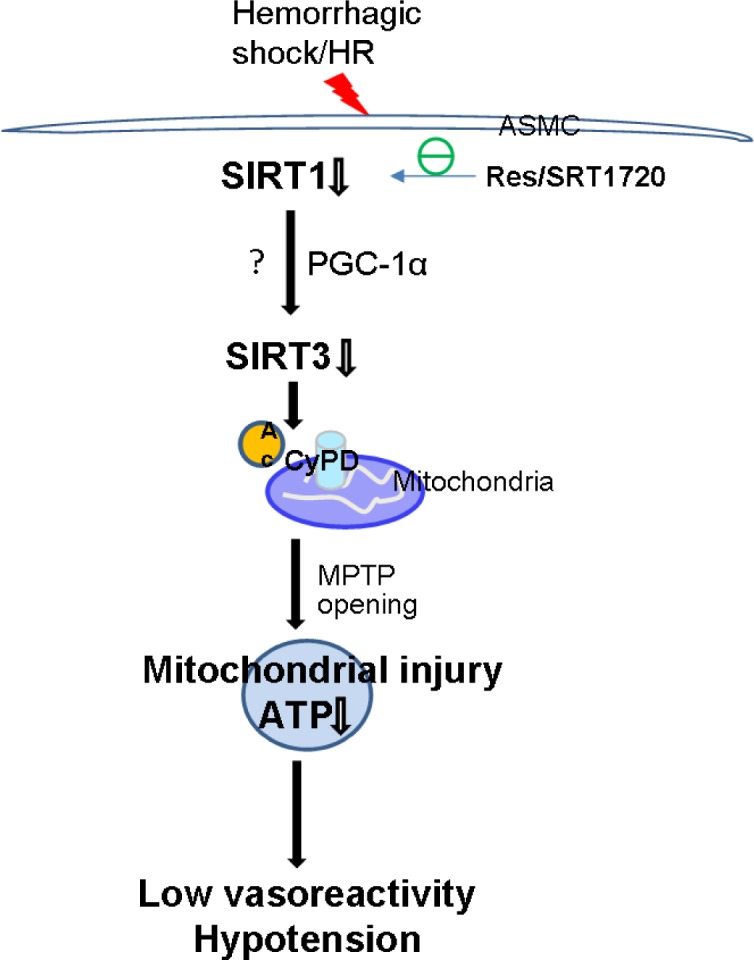
Schematic illustration of the mechanism underlying low vasoreactivity and hypotension in severe shock SIRT1/SIRT3 pathway regulates mPT pore opening and mitochondrial function via deacetylation of CyPD in ASMCs. Activation of SIRT1 by Res or SRT1720 improves low vasoreactivity and refractory hypotension following severe shock and other conditions connected with cytopathic hypoxia.

SIRT1 can be activated by resveratrol, and SIRT1 activity is necessary for resveratrol-mediated mitochondrial biogenesis [13, 32, 41]. Resveratrol treatment can restore SIRT1 activity, and improve SIRT3 deacetylase activity in ASMCs during severe hemorrhagic shock. In addition, inhibition of SIRT1 with EX527 abrogates the effect of Res on SIRT3 deacetylase activity, indicating a critical role of SIRT1 in the regulation of SIRT3 activity in vascular cells. Indeed, the results in the experiments where the specific SIRT1 activator SRT1720 is applied confirmed the role of SIRT1 in the regulation of SIRT3 activity. Further investigation is required to determine whether SIRT1 directly targets SIRT3 to regulate mitochondrial function or indirectly *via* other mediators. Activation of SIRT1 also prevents persistent opening of mPT pore and the decline of intracellular ATP, and improves vasoreactivity and MAP. Our results are consistent with previous findings that resveratrol treatment protects against mitochondrial injury and improves survival in rats with severe hemorrhagic shock and other cardiovascular conditions [42, 43]. Based on these data, we propose that SIRT1/3 play a protective role against mitochondria injury and dysfunction in ASMCs and thereby preserves vasoreactivity during severe hemorrhagic shock (Figure 5).

In summary, we have provided evidence that mitochondrial injury in ASMCs during severe hemorrhagic shock is associated with decreased protein levels and attenuated deacetylase activity of SIRT1/3. Modulation of mPT by SIRT1/3-mediated deacetylation of CyPD in vascular smooth muscle cells is required for the preservation of vasoreactivity. These novel findings suggest that SIRT1/3 is a promising therapeutic target for refractory hypotension following severe hemorrhagic shock.

## MATERIALS AND METHODS

### Animals

238 male and female rats of Wistar strain (180-220 g, 8-10 weeks old) were purchased from the Experimental Animal Center of the Southern Medical University (Guangzhou, China) and housed individually under controlled environmental conditions with a 12-hour light/dark cycle and ad libitum access to pellet food and water throughout the study. All animal experiments were conducted in the Key Laboratory of Shock and Microcirculation (Southern Medical University, Guangzhou, China), in accordance with the Chinese National Guidelines for the Use and Care of Experimental Animals, and the protocols were approved by Experimental Animal Ethics Committee of the Southern Medical University.

### Severe hemorrhagic shock model

The rats were anesthetized by the administration of a mixture of 13.3% urethane and 1% chloralose (0.6 ml/100 g i.p.). Severe hemorrhagic shock was induced as previously described [24]. Briefly, both sides of femoral arteries were cannulated using polyethylene catheters (PE-50) to measure mean arterial pressure and withdraw blood, and the left femoral vein was cannulated for the administration of drugs and resuscitation. To induce severe hemorrhagic shock with a target mean arterial pressure (MAP) of 30 mmHg, blood was withdrawn from the arterial catheter within a 10-min period under continuous blood pressure monitoring.

The spinotrapezius muscles were isolated from rats and prepared for intravital microscopy using the technique described by Gray [44]. Briefly, unbranched, third-order arterioles (diameter range 20-30 μm) were selected at random and videotaped at 2000 frames/s using a CR5000X2 high-speed camera (Optronis, Germany). The vasoreactivity was determined by topical application of norepinephrine (NE) until the threshold concentration was reached, i.e., the concentration that produced complete cessation of arteriolar blood flow in no less than 20 s. This threshold concentration was used as a measure of arteriolar reactivity. Throughout the surgical preparation and experimental procedures, the exposed muscle was continuously superfused with balanced Krebs solution containing 132 mM NaCl, 5 mM KCl, 2 mM CaCl2, and 1.2 mM MgSO4 (pH adjusted to 7.4 with NaHCO3 at 37°C).

Res (30 mg/kg) and SRT1720 (20 mg/kg, body weight, iv) were administered 2 h post bleeding with or without the administration of EX-527 before bleeding.

### Transmission electron microscopy

Freshly isolated mesenteric arterioles were quickly fixed with 2.5% glutaraldehyde for 24 h. After three 10-min washes with 0.13 M PBS, the tissue was postfixed in 1% OsO4 for 2 h, dehydrated in a graded series of ethanol (50%, 70%, 80%, 90%, and 100% for 10 min each), and then incubated with tert-butoxide for 10 min. Samples were dried with CO_2_, coated with gold (Au) using ion sputter coater, and viewed and imaged with a transmission electron microscope (H-7500, Hitachi Company, Japan).

The extent of ultrastructural damage was quantified by using Flameng's score [45]. For each sample, 5 field views were randomly selected and 20 mitochondria were selected per field view. The criteria for mitochondrial damage are shown in supplemental Table 1.

### ASMC isolation

Arteriolar smooth muscle cells (ASMCs) were isolated from mesenteric arterioles (the second A2 branch) of the rats [2]. Briefly, after the dissociation with 0.3 mg/ml papain and 1 mg/ml collagenase, cells were maintained at 4°C in HPSS buffer (130 mM NaCl, 5 mM KCl, 1.2 mM MgCl_2_, 0.1 mM CaCl_2_, 10 mM HEPES, and 10 mM glucose). The dissociated cells were identified as smooth muscle cells under inverted phase contrast microscopy and indirect immunohistochemical staining with *α*-smooth muscle actin monoclonal antibody [46].

### Measurement of intracellular ATP

ASMC ATP levels were measured by a luciferase-based assay (CellTiter-Glo,** Promega, Madison, WI, USA) according to the manufacturer's protocols. The luminescence was recorded in an automatic microplate reader (SpectraMax M5; Molecular Devices, Sunnyvale, CA, USA).

### Western blotting

Total protein was isolated from arterioles or ASMCs by homogenization in cold RIPA lysis buffer containing protease inhibitors and phosphatase inhibitors (Roche). Equal amounts of proteins were separated by SDS-PAGE and transferred to a polyvinylidene difluoride membrane. After blocking in 5% bovine serum albumin (BSA) in Tris-buffered saline/Tween-20 (50 mM Tris, pH 7.5, 500 mM sodium chloride, and 0.05% Tween-20) for 1 h at room temperature, the membranes were incubated overnight at 4°C with primary antibodies against SIRT1 or SIRT3 (Cell Signaling Technology, Danvers, MA, USA), and GAPDH or β-actin (Santa Cruz Biotechnology, Santa Cruz, CA, USA). After incubation with a secondary antibody conjugated to horseradish peroxidase (Santa Cruz Biotechnology) for 1 h at room temperature, immunoreactivity signals were detected by chemiluminescence using ECL detection reagent (Advansta Westernbright ECL, USA), visualized using a chemiluminescence detection system (Image Station 4000R, KODAK, USA), and analyzed using Quantity One software (version 4.52).

### SIRT1/3 deacetylase activity assay

SIRT1 deacetylase activity was detected using the Cyclex SIRT1/SIRT2 and SIRT3 Deacetylase Fluorometric Assay Kit (Cyclex, Nagano, Japan). Briefly, mesenteric arterioles (100 mg) or ASMCs were homogenized in 500 μL of immunoprecipitation buffer (T-PER^®^ Tissue Protein Extraction Reagent 78510, Pierce, USA). After immunoprecipitation of SIRT1/3, the final reaction mixtures (50 μL) contained 50 mM Tris-HCL (pH 8.8), 4 mM MgCL_2_, 0.5 mM DTT, 0.25 mA/mL Lysyl endopeptidase, 1 μM Trichostatin A, 200 μM NAD^+^ and 5 μL of extraction. The fluorescence intensity (ex. 340 nm, em. 460 nm) was measured using an automatic microplate reader (SpectraMax M5; Molecular Devices, Sunnyvale, CA, USA).

### Immunofluorescence

Freshly isolated cells were plated on the Petri dish and stained with MitoTracker Red dye CMXRos (300 nM) at room temperature for 15 min. Next, the cells were fixed in 4% paraformaldehyde, permeabilized in blocking solution (5% BSA in PBS) containing 0.2% triton X-100 for 1 h, and then incubated with primary antibody for SIRT1 or SIRT3 overnight at 4°C. Cells were washed with PBS and incubated with secondary antibodies (Invitrogen Alexa Fluor® 488 goat anti-rabbit IgG or Alexa Fluor 647 goat anti-mouse IgG) for 2 h at room temperature. The nuclei were counterstained with DAPI (1 μg/ml). Stained cells were examined under confocal microscope (LSM710, ZEISS, Germany). All data presented are representative of six independent rats.

### Determination of mitochondrial permeability transition pore opening

Mitochondrial permeability transition (mPT) pore opening was directly assessed by co-loading cells with calcein-AM and CoCl_2_ as previously described [47]. The changes in calcein-AM fluorescence signal were analyzed by flow cytometry (excitation filter 488 nm and emission filter 515 nm).

### Determination of mitochondrial membrane potential

Mitochondrial membrane potential (MMP) was determined by monitoring fluorescence aggregates of JC-1 (tetrachloro-1, 1′, 3, 3′ - tetraethyl - 6′, 6, 5′, 5- benzamidazolocarbocyanin iodide). Regions of high mitochondrial polarization were indicated by red fluorescence due to J-aggregate formation by the concentrated dye. Depolarized regions were indicated by the green fluorescence of the JC-1 monomers. Freshly isolated ASMCs were incubated with 5 μg/ml JC-1 for 20 min at 37°C in a humidified incubator, then washed twice with PBS, and the fluorescent ratios (for red fluorescence: excitation at 490 nm and emission at 590 nm; for green fluorescence: excitation at 490 nm and emission at 527 nm) were detected by flow cytometry (BD FACSverse, USA).

### Statistical analysis

Statistical analyses were performed using SPSS (version 17.0). All values were presented as mean and standard error (SE). One-way or two-way analysis of variance (ANOVA) was used to compare the differences between two groups or among multiple groups, respectively, followed by Tukey's post hoc tests. P values less than 0.05 were considered statistically significant. *n* represented the number of animals.

## SUPPLEMENTARY MATERIAL TABLE


