# Supplementary material for: Activation of sirtuin 1/3 improves vascular hyporeactivity in severe hemorrhagic shock by alleviation of mitochondrial damage
Source: Oncotarget. 2015 Oct 12;6(35):36998–7011. doi: 10.18632/oncotarget.6076 (PMC4741911; doi:10.18632/oncotarget.6076)
Supplement: Supplementary file 1 [file oncotarget-06-36998-s001.pdf]

Activation of sirtuin 1/3 improves vascular hyporeactivity in severe hemorrhagic shock by alleviation of mitochondrial damage

Supplementary Material

Supplemental Table 1

Score	Grading standard
0	Normal structure with well-preserved mitochondrial granules
2	Normal structure but granules absent
3	Swollen mitochondria with clarification of the matrix
4	Disruption of mitochondrial crests with clarification as well as condensation of the matrix
5	Disruption of the crests and loss of integrity of the mitochondrial inner and outer membrane
